# 

**Published:** 1852-02

**Authors:** 


### CONTENTS

OF THE

### MEDICAL EXAMINER.

NEW SERIES—NO. LXXXVI.—FEBRUARY, 1852.

### ORIGINAL COMMUNICATIONS.

Cases of Synovial Articular Inflammation of the Knee, treated successfully with Urate of Ammonia. By W. E. Horner, M. D., Prof. of Anatomy in the University of Pennsylvania, Senior Surgeon of the St. Joseph's Hospital, etc., - - - 69

A Case of Curious Cutaneous Disease. By S. Weir Mitchell, M. D., of Philadelphia, - - - - - - - 74

Extracts from a Lecture "On the present position in Europe of some of the most interesting and important points of Modern Surgery," recently delivered as an Introductory Discourse. By Thomas D. Mütter, M. D., Professor of Surgery in Jefferson Medical College, Philadelphia. (Continued,) - - 76

Fecundity of a Free-Martin. By Joseph Moore, of the Jefferson Medical College, and of Cumberland Co., New Jersey. (Communicated by Prof. Dunglison,) - - - 82

### BIBLIOGRAPHICAL NOTICES.

A Practical Treatise on the Diseases of the Lungs and Heart, including the Principles of Physical Diagnosis. By Walter Hayle Walshe, M. D., Professor of the Principles and Practice of Medicine, and of Clinical Medicine, in University College, London; Physician to the University College Hospital, - 83

The Transactions of the American Medical Association. Instituted 1847, - - - - - - - 93

The Microscopic Anatomy of the Human Body in Health and Disease. Illustrated with numerous drawings in colors. By Arthur Hill Hassall, M. D., Author of a "History of Fresh Water Algæ," Fellow of the Linnæan Society, &c. &c. With additions to the text and plates, and an introduction containing instructions in Microscopic Manipulations. By Henry Vanarsdale, M. D., - - - - - - - 105

Homœopathy; an Examination of its Doctrines and Evidences. By Worthington Hooker, M. D., - - - - 107

A Memoir of Samuel George Morton, M. D. By Charles D. Meigs, M. D. Read before the Academy of Natural Sciences, November 6th, 1851, - - - - - - 109

A Review of Materia Medica, for the use of Students. By John B. Biddle, M. D., formerly Professor of Materia Medica in the Franklin Medical College of Philadelphia, &c. &c., - - 109

### EDITORIAL.

Death of Dr. Thomas Mackie Smith, - - - - 110

Sydenham Society of London, - - - - - 110

Instruction in Microscopy, - - - - - - 110

### MEDICAL NEWS.

Homœopathy, - - - - - - - - 111

Death of Dr. James Cameron, - - - - - 114

Death of Dr. Sydney A. Doane, - - - - - 114

Death of Dr. James Arthur, - - - - - 114

The Royal Society of England, - - - - - 114

The London Epidemiological Society, - - - - 115

Arsenical Poisoning, - - - - - - - 115
Death of Priessnitz, - - - - - - 116
The French Revolution, - - - - - - - 116
The London Medical Times and London Medical Gazette, - 116
Annual Public Meeting of the Academy of Medicine of Paris, - 117

### RECORD OF MEDICAL SCIENCE.

### SURGERY.

Gonorrhœa, - - - - - - - 117

### MATERIA MEDICA AND THERAPEUTICS.

Comparative Value of Cod-Liver Oil and Fish Oil mixed with Iodine, 118
Extraction of Balsam of Peru, - - - - - 118
Iodine rendered soluble by Syrup of Orange-peel and Tannin, - 119
Use of Tannate of Alumina, - - - - - - 119

### PATHOLOGY AND PRACTICE OF MEDICINE.

Prurigo of the Genital and Anal Regions, - - - - 120
Hydatids of the Liver Evacuated Externally. By M. Lafforgue, - 120
Case of Paraplegia Successfully Treated by the Ergot of Rye, - 121
Phosphorus in Beriberi and other Diseases. By W. G. Maxwell, Esq., M. D., Garrison Surgeon, East Indies, - - - 121
Tying of the Carotid Artery, followed by Softening of the Brain, and Death, - - - - - - - - 122
Diet in Protracted Fevers. By E. B. Haskins. M. D., - - 122
Sesquicarbonate of Ammonia in Lepra and Psoriasis, - - 124

### ANATOMY AND PHYSIOLOGY.

Muscular Contraction—Cadaveric Rigidity—French and American Experiments, &c., - - - - - - - 124
On the Endosmotic action of Medicines, - - - - - 130
Observations on the Sounds of the Heart. By Richard Brown, Esq., M. R. C. o,, L. A. C., &c., Cobham, - - - 135

### OBSTETRICS.

On Relaxation of the Symphysis Pubis after Labor, - - 136

